# Antifungal and plant growth promotion activity of volatile organic compounds produced by *Bacillus amyloliquefaciens*

**DOI:** 10.1002/mbo3.813

**Published:** 2019-03-24

**Authors:** Yuncheng Wu, Jinyan Zhou, Chengguo Li, Yan Ma

**Affiliations:** ^1^ Institute of Agricultural Resources and Environment Jiangsu Academy of Agricultural Sciences Nanjing China; ^2^ School of Animal, Rural and Environmental Sciences Nottingham Trent University Nottingham England

**Keywords:** *Bacillus amyloliquefaciens*, *F. oxysporum* f.sp. *niveum*, root colonization, volatile organic compounds, watermelon

## Abstract

*Fusarium* wilt of watermelon, caused by *F. oxysporum* f.sp.* niveum* (FON), is a devastating disease that causes extensive losses throughout the world. Five bacterial strains (L3, h, β, b, and L) isolated from the watermelon rhizosphere showed antagonistic activity against FON during in vitro tests. Strain L3 produced diffusible and volatile organic compounds (VOCs) which showed the strongest antifungal activity. *Arabidopsis thaliana *plantlets exposed to VOCs produced by strain L3 showed a 2.39‐fold increase in biomass, 1.40‐fold increase in primary root length, and 5.05‐fold increase in number of lateral roots. Confocal laser scanning microscope showed that the GFP‐labeled strain L3 could colonize along the elongation and differentiation zones of watermelon roots. In greenhouse pot experiments, the biocontrol efficiency of strain L3 against fusarium wilt of watermelon was up to 68.4% in comparison with the control treatment. In addition, inoculation of the strain L3 resulted in a 23.4% increase in plant fresh weight. Based on 16S rDNA sequence analysis, the strain L3 was identified as *Bacillus amyloliquefaciens *L3. Fourteen VOCs produced by strain L3 were identified through GC‐MS analysis. Of nine VOCs tested, 2‐nonanone and 2‐heptanone were proved to have strong antifungal properties. Acetoin and 2,3‐butanediol were found to promote plant growth. The results suggested *B. amyloliquefaciens *L3 was a potential biocontrol agent, and that VOCs produced by *B. amyloliquefaciens *L3 play important roles in the process of biocontrol and plant growth promotion.

## 1 INTRODUCTION

Watermelon is an important fruit crop that contributes to food and economic security in addition to human nutrition. In China, continuous mono‐cropping of watermelon has become a popular practice to meet the growing consumer demands. This continuous cultivation also led to the establishment of fusarium wilt and other soilborne diseases which threaten watermelon production. *Fusarium* wilt of watermelon is caused by *Fusarium oxysporum* f. sp. *niveum* (FON) (Ling, Deng, & Song, 2014; Zhang, Xu, & Liu, 2015). The pathogen can survive in soil in the absence of host for up to 10 years as chlamydospores that makes the traditional control method ineffective, such as crop rotation (Liu, Chen, & Zhao, 2018). There are no commercial resistant cultivars against Fusarium wilt. Chemical soil fumigation is one of the leading methods for controlling *Fusarium* wilts (Sun, Song, & Fu, 2015). However, soil fumigation with chemicals is known to have broad biocidal activity, and detrimental effects were found not only on target pathogens but also on non‐target microorganisms and the fumigation chemicals themselves could be extremely dangerous to humans (Yan, Wang, & Li, 2017). The application of biological control agents (BCA) has been recognized as an environmentally friendly and sustainable method to reduce the effects of plant diseases (Raza, Wang, Wang, & Wu, 2016; Raza, Wei, Wei, & Ling, 2016; Saravanakumar, Li, & Yu, 2013).

Rhizosphere soils from healthy plants which survived in a field infested with phytopathogens are a good source for the isolation of biocontrol agents (Huang, Wei, & Tan, 2013). In recent decades, many biocontrol agents have been successfully isolated from rhizosphere niches, such as *Bacillus* spp., *Pseudomonas* spp., *Trichoderma* spp., *Streptomyces* spp., and effectively used for the control of *Fusarium* wilt in many different commercial crops (Faheem, Raza, & Wei, 2015). Among these promising BCAs, *Bacillus* spp. are well‐known for their inherent property to produce spores and for their resistance to extreme conditions (Shafi, Tian, & Ji, 2017). *Bacillus subtilis *SQR9 isolated from the rhizosphere of cucumber, showed strong antagonism against *F. oxysporum* f.sp. *cucumerinum *(Cao, Zhang, & Ling, 2011). Akarm, Mahboob, and Javed (2013) showed that *B. thuringiensis* strain 199 can protect tomato plants against *Fusarium* wilt. The genus *Bacillus* spp. were found to be well adapted to the rhizosphere of watermelon. However, relative few biocontrol agents belonging to *Bacillus *spp. are used to control the Fusarium wilt of watermelon (Raza, Yuan, & Ling, 2015; Zhao, Wang, & Liang, 2018). Thus, more studies are still needed to explore biocontrol agents belonging genus *Bacillus* to strengthen the range of weapons available for the biocontrol of Fusarium wilt of watermelon.

*Bacillus *strains exhibit various biological control mechanisms, such as production of a wide spectrum of antibiotics, synthesis extracellular enzymes, competition for nutrients and niches, and induction of systemic resistance in plants against pathogens (Chowdhury, Hartmann, & Gao, 2015; Santoyo, Orozco‐Mosqueda, & Govindappa, 2012). Among these mechanisms, previous research has shown that root colonization ability is a prerequisite for biological control agent's activity (Mendis, Thomas, Schwientek, & Salamzade, 2018). Cavaglieri, Orlando, and Rodriguez (2005) reported that the biological control efficiency of plant pathogens was directly related to the root colonization ability of biological agents. Zhang, Wu, He, and Li (2011) demonstrated the critical importance of the colonization of *B. subtilis *N11 on banana roots to stop the pathogen invading by using a GFP‐tagged *B. subtilis* N11. Therefore, it is essential to evaluate root colonization ability when searching promising BCAs.

A variety of antifungal compounds, such as lipopeptides which belong to the iturin, surfactin, and fengycin group, are well‐documented as being produced by *Bacillus *spp. (Kim, Kang, Kwon, & Seo, 2015). In addition to these non‐volatile antimicrobial compounds, increased research effort has been devoted to revealing the function of VOCs in recent years (Jamalizadeh, Etebarian, Aminian, & Alizadeh, 2011; Raza, Wang et al., 2016; Raza, Wei et al., 2016). VOCs are low–‐molecular‐weight compounds that easily evaporate at normal temperature and pressure (Gotor‐Vila, Teixidó, Di, & Usall, 2017). Previous studies have reported that the VOCs produced by *Bacillus *spp. can inhibit the growth and spore germination of several plant fungal pathogens (Tahir, Gu, Wu, & Raza, 2017; Tahir, Gu, Wu, & Niu, 2017). In addition to antifungal activity, the VOCs produced by biocontrol strains can also improve plant growth and induce resistance in plants against plant pathogens (Gao, Zhang, Liu, & Han, 2017; Ryu, Farag, Hu, & Reddy, 2003). The effective components of VOCs vary between different biocontrol strains. For example, Gao et al. (2017) found four anti‐fungal VOCs, pyrzine017, benzothiazole, 4‐chloro‐3methyl, and phenol‐2,4‐bis (1,1‐dimethylethyl), released by *B.velezensis* ZSY‐1. Among the total of 36 VOCs detected from *B. amyloliquefaciens* NJN‐6, 11 compounds can completely inhibit fungal growth (Zou, Li, & Yu, 2010). Three compounds, Albuterol and 1,3‐propanediole produced by *B. subtilis* strain SYST2, and 2‐pentylfuran produced by *B. megaterium* XTBG34 each additionally demonstrate plant growth promotion activity (Tahir, Gu, Wu, & Raza, 2017; Tahir, Gu, Wu, & Niu, 2017; Wu, Yuan, Raza, & Shen, 2014).

In this study, five (L3, h, β, b, and L) potential biocontrol strains of *Bacillus* were isolated from the healthy watermelon plants which survived in a field heavily infested with FON. The objectives of the study were to: (a) isolate Bacillus with antifungal activity against FON, (b) evaluate antifungal and plant‐growth promotion effect of VOCs produced by the isolated strains, (c) evaluate the root colonization ability of the selected biocontrol strain, (d) evaluate the biocontrol efficiency and plant growth effect by performing greenhouse pot experiments, and (e) discover the components and functions of VOCs secreted by the selected strain.

## 2 MATERIALS AND METHODS

### 2.1 Microorganisms and growth conditions

The culture of *F. oxysporum* f. sp. *niveum* (FON) used in this study was originally isolated from infected stems collected from watermelon fields located in Jiangsu, China by the method described by Chang, Ling, Chen, and Huang (2015). FON was pathogenic to watermelons in in vivo experiments. Stock cultures were maintained on potato dextrose agar (PDA) plates at 4°C. Pre‐cultures were established by transferring a stock agar plug containing mycelia onto fresh PDA plates and incubating for 4 days at 28°C.

### 2.2 Isolation and screening of bacteria for biocontrol activity

The rhizosphere soils were sampled from healthy watermelon plants that had survived in a field with a history of *Fusarium wilt *in Jiangsu province of China. The serial‐dilution‐pour method was used to isolate rhizosphere bacteria on nutrient agar (Beef extract 3.0 g/L, peptone 5.0 g/L, sodium chloride 3.0 g/L, and agar 20.0 g/L). The bacterial strains were further purified and then screened for antagonistic activity toward FON using dual culture technique. Briefly, a 3 mm agar plug from the edge of a 4‐day‐old culture of FON on PDA was placed in the middle of fresh PDA plate. The bacterial strains were spot inoculated in the edge of the plate and incubated at 28°C. The PDA plate inoculated only with FON was used as control treatment. Each treatment was repeated three times. The diameter of FON in each plate was then recorded after 5 and 10 days.

### 2.3 Antagonistic effects of VOCs on growth of FON

The antagonistic effects of VOCs produced by the selected bacterial strains (L3, h, β, L, and b) isolated above, were measured according to the method described by Wu et al. (2014). The Petri dish containing modified Murashige‐Skoog (MS) culture medium (0.5% TSB and 2% agar) inoculated with the isolated strains was covered with another Petri dish containing PDA inoculated with a 6 mm diameter plug of FON. Then the two dishes were sealed with Parafilm to obtain a double‐plate chamber. The average distance between MS and PDA agar surface was 1.5 cm. The double‐plate chamber was incubated at 28°C for 4 days. The double‐plate chamber without bacterial strains were used as control. The experiment was repeated three times.

### 2.4 Plant growth promotion by VOCs

**Figure A1:**
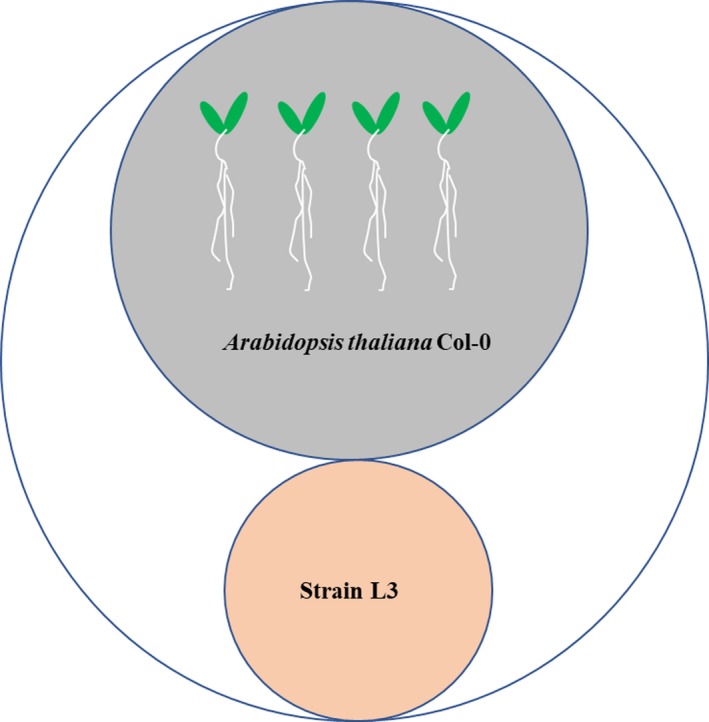
Schematic representation of the experimental setup for *Arabidopsis thaliana *growth promotion by VOCs

*Arabidopsis thaliana *Col‐0 seeds were surface sterilized in 70% (v/v) ethanol for 30 s and afterwards incubated for 5 min in sodium hypochlorite solution, then rinsed with sterile water for four times. Seeds were placed on Petri dishes containing 0.5 × MS for 2 days at 4°C and then germinated for 2 days on Petri dishes containing 0.5 × MS, 0.8% sucrose and 0.6% bacto agar. Then, five plantlets were transferred to 90 mm diameter Petri dishes containing 0.5 × MS, 0.8% sucrose and 1% bacto agar. Each of the five selected bacterial strains was pre‐cultured in a 60 mm diameter Petri dish containing MS, 0.5% TSB, and 2% bacto agar for 24 hr at 30°C. Then two Petri dishes containing plantlets and bacterial strains were put into a 150 mm diameter plate and sealed with Parafilm and placed vertically in a growth chamber at 22°C, 16 hr light/8 hr dark (Appendix Figure A1). Each treatment had three replicates. Plant fresh weight, primary root length, and lateral root number were measured after 7 days incubation.

### 2.5 Construction of GFP‐tagged L3 and root colonization assay

Based on the results of 2.3–2.5 section, the strain L3 was chosen for further studies. The gfp‐marked shuttle vector, pHAPII (GenBank accession number HM151400), was used to construct the GFP‐tagged L3 by the method described by Cao et al. (2011). A GFP‐tagged L3 mutant that emitted green fluorescence was chosen and used in the root colonization assay.

Watermelon “Sumi 8” were surface‐sterilized by immersing in sodium hypochlorite solution (2%, v/v) for 15 min and rinsed five times in sterilized distilled water. The seeds were placed on moist filter papers in a petri dish under sterile conditions for germination at 28°C. After 3 days, the germinated seeds were then sown in soilless culture medium (3:2:3:2 mix of fermentation bed farming material; vermiculite; perlite; peat) at 28°C with 16 hr light/8 hr dark. Then the seedlings with 1–2 leaves were used for the subsequent studies.

A hydroponic system was used for colonization assays as described by Zhang et al. (2011). The watermelon seedlings were collected and gently washed to remove the adhered substrate. The seedlings were placed into 50‐ml flasks (one seedling in each flask) containing 50 ml of liquid 1/2 MS medium at 28°C. A 1 ml suspension of GFP‐tagged L3 (10^8 ^cfu/ml) was added to each flask of hydroponic medium. The culture conditions were the same as described above. The root samples were collected at 48 hr and 96 hr. Part of each root sample (0.1 g) was ground in a mortar with 0.9 ml sterilized distilled water until a fine homogenate was obtained. The suspension was diluted, then plated on modified LB medium (20 μg/ml, kanamycin) and colony‐forming units were counted after incubating at 37 ℃ for 2 days. The patterns of GFP‐tagged L3 colonization of watermelon roots were examined by a Confocal Laser Scanning Microscope (Leica Model TCS SP2, Heidelberg, Germany) as described by Huang, Zhang, Yong, and Yang (2012). Roots without inoculation with the GFP‐tagged L3 served as control. Each treatment was repeated three times.

### 2.6 Biological control and plant growth promotion activity of the strain L3

The ability of strain L3 to suppress *Fusarium *wilt of watermelon was investigated in an FON‐infested soilless growth substrate. Treatments included: a soilless growth substrate inoculated with FON as a control (CK), and soilless growth substrate inoculated with FON and the strain L3 (L3). Each treatment included 30 watermelon seedlings. The spore suspension of FON (10^6^ CFU/ml) was first drenched into the growth substrate, followed by a suspension of strain L3 (10^9^ CFU/ml). The final concentration of FON (10^5^ CFU/g) and strain L3 (10^8^ CFU/g) in the growth substrates. Watermelon seedlings were then transplanted in the substrate trays (450 × 20 cm), and then grown at 16 hr light/8 hr dark at 28°C. Plants were watered as required for plant growth and disease development. Disease incidence was recorded after 28 days when symptoms of Fusarium wilt was starting to appear on the watermelon plants, and continued for another 8 days. Incidence of Fusarium wilt was calculated by using the formula:

$$\text{Disease}\;\text{incidence}\left(\%\right)=\frac{\text{Number}\;\text{of}\;\text{diseased}\;\text{plants}}{\text{Total}\;\text{number}\;\text{of}\;\text{plants}}\times \text{1}00$$

The growth substrate samples near to watermelon root rhizosphere were collected at 0, 20, and 36 days after transplanting and stored at −20°C. The population of FON in the growth substrate samples were determined by real‐time PCR following the method described by Zhao, Liu, and Ling (2014).

The plant‐growth promotion ability of the strain L3, was assessed in pot experiment with a non‐infested growth substrate as described above. Briefly, strain L3 was used as a drench treatment, and control pots received an equal volume of sterile distilled water. Each treatment consisted of three replicate pots (one plant per pot). After 20 days of transplanting, plant growth was determined by weighing individual plants.

### 2.7 Identification of VOCs by SPME‐GC‐MS

Solid‐phase micro‐extraction (SPME) technique was used to collect VOCs produced by the strain L3 (Wu et al., 2014). Briefly, a 150 ml vial containing 50 ml modified MS culture medium (1.5% sucrose, 0.4% TSB and 2% agar) was inoculated with 200 μl cells suspension of the strain L3 (10^8^ CFU/ml) and incubated at 28 ℃ for 2 days. Vials with only 50 ml culture medium were used as control. Then the vial was placed in a water bath (50°C) for 30 min with the SPME fiber (divinylbenzene‐carboxene‐PDMS, 50/30 μm) inserted into the headspace. The SPME fiber was desorbed at 220°C for 1 min in the injection port of GC/MS with a RTX‐5MS column (30 m, 0.25‐mm inside diameter, 0.25 μm). GC‐MS run was performed for 24 min. The initial oven temperature was 33°C, held for 3 min, ramped up first at a rate of 10°C/min to 180°C, and then ramped up at a rate of 40°C/min to 240°C, and held for 4 min. The mass spectrometer was operated in the electron ionization mode at 70 eV, a source temperature of 220°C, and with a continuous scan from 50 *m/z* to 500 *m/z*. The retention time and mass spectra of VOCs were compared with those in the NIST/EPA/NIH Mass Spectrometry Library. Then the VOCs profiles of the strain L3 were compared with the respective control, and only the peaks resulted from the L3 inoculated vials were identified.

### 2.8 Verification of synthetic compounds against FON

Among the identified VOCs, nine standard compounds were purchased from the reagent company (Appendix Table A1). The antifungal activity of the standard compounds was assessed using the I‐plate system described by Yuan, Raza, Shen, and Huang (2012). The I‐plate was prepared with PDA on one side and inoculated with a FON plug (4 mm). Then 50 μl of each standard compound was added to the other side. The I‐plate was sealed with Parafilm and incubated at 28°C. The I‐plates added with methanol or distilled water were used as control. The colony diameter of FON was recorded after 4 days incubating. The experiment was repeated three times.

### 2.9 Plant growth promotion activities of synthetic compounds

The plant growth promotion activities of the nine compounds were measured by the modified method described by Ryu et al. (2003) and Zou et al. (2010). Briefly, three 2‐day‐old germinated *Arabidopsis thaliana *Col‐0 seedlings were transferred to the one side of the I‐plate containing 0.5 × MS, 0.8% sucrose and 1% bacto agar. Then, the nine synthetic compounds were diluted separately in alcohol, and 20 μl of the resulting suspension was applied to a sterile filter paper disk on the other side of the I‐plate. A total of 10 μg, 100 μg, 500 μg and 1,000 μg doses of each synthetic compounds were tested. Each treatment was repeated for three times. The fresh weight of the *Arabidopsis thaliana *Col‐0 seedlings was measured after 10 days.

### 2.10 Characterization of strain L3

The biochemical and physiological characteristics of strain L3 were tested according to the method described by Wu et al. (2014). The genomic DNA of the strain L3 was extracted by using the E.Z.N.A. Bacterial DNA kit (OMEGA Ltd.). The primer pair (F：5′‐ AGAGTTTGATCCTGGCTCAG‐3′; R：5′‐AAGTCGTAACAAGGTA‐3′) was used to amplify the 16S rRNA gene of the strain L3. Then the 16S rRNA gene was sequenced and Blast searched against the NCBI database. The sequences of its close relatives were used to construct a neighbor‐joining phylogenetic tree using MEGA 4.0.

### 2.11 Statistical analysis

The data were assessed with one‐way ANOVA. Duncan's multiple‐range test was applied when one‐way ANOVA revealed significant differences (*p* ≤ 0.05). All statistical analyses were performed with the SPSS ver. 13.0 statistical software (SPSS, Chicago, IL).

## 3 RESULTS

### 3.1 Isolation of bacteria for biocontrol activity

**Figure 1:**
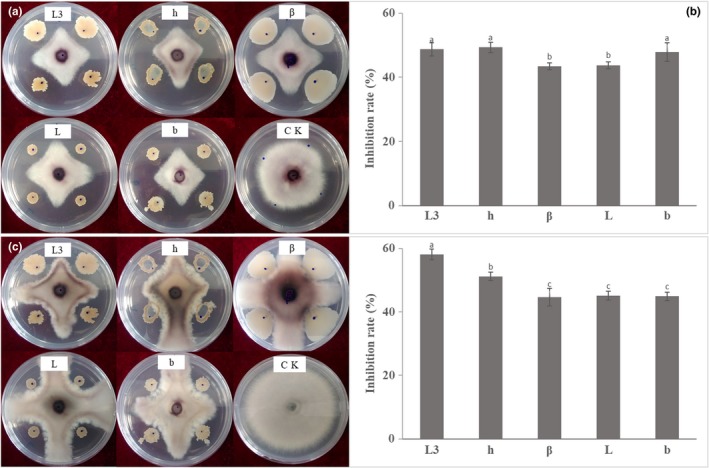
Antifungal activity of the bacteria (L3, h, β, L, and b) isolated from watermelon rhizosphere soil against *Fusarium oxysporum* f. sp. *niveum* (FON). (a) Antagonism pattern after 5 days dual culture assay. (b) Inhibition rate of the isolated strain after 5 days. (c) Antagonism pattern after 10 days dual culture assay. D: Inhibition rate of the isolated strain after 10 days. Lowercase letters above the columns indicate a significant difference at *p* < 0.05

Among the bacteria isolated from the soil samples, five strains that showed antagonistic activity against FON were named as L3, h, β, L, and b (Figure 1), respectively. After 5 days of incubation, the antifungal activities of these five strains were similar. The inhibition rates were 48.71%, 49.35%, 43.37%, 43.80%, and 47.86%, respectively. After 10 days of incubation, the inhibition rates of these strains were 58.12%, 51.21%, 44.67%, 45.08%, and 44.91%, respectively. Strain L3 showed the strongest antifungal ability out of the five strains.

### 3.2 Antagonistic effects of VOCs on growth of FON

**Figure 2:**
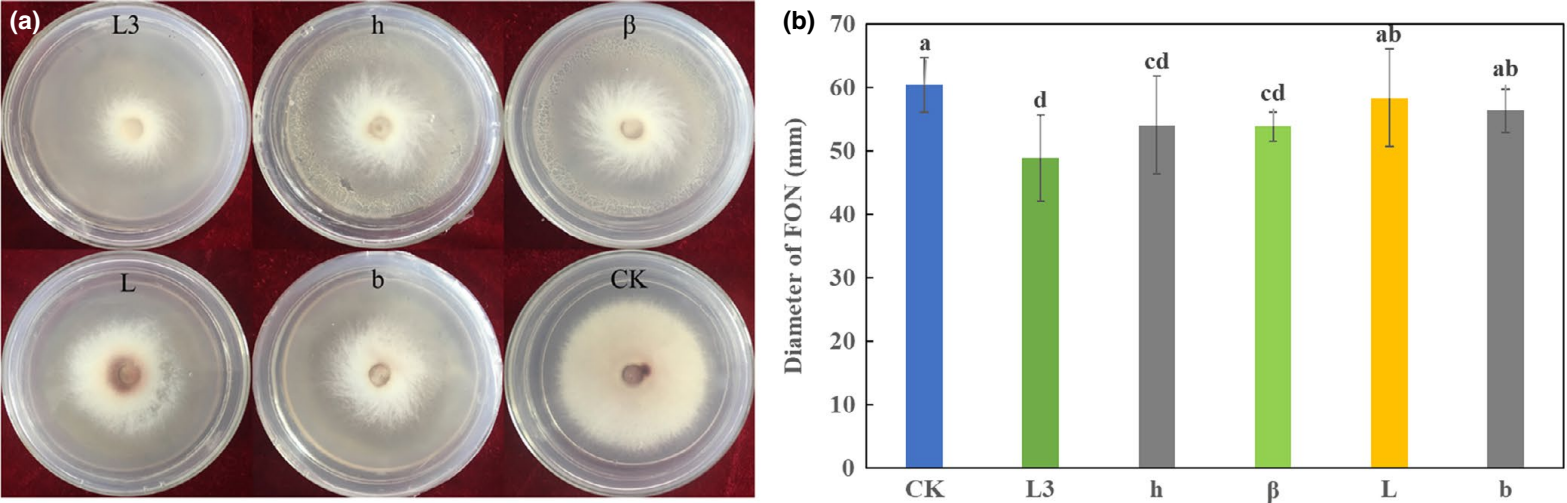
Antifungal volatile activity of isolated strains (L3, h, β, L, and b) on double‐dish chamber. (a) Mycelial growth of FON was inhibited in the presence of the bacteria streaked in the bottom dish. (b) Diameter of mycelial growth in the presence of VOCs after culture for 120 hr. Lowercase letters above the columns indicate a significant difference at *p* < 0.05

All five strains showed significant inhibitory effects against FON mycelial growth during the bioassay without direct contact, although the VOCs produced by these strains could not completely inhibit the FON mycelia growth (Figure 2a). The morphology of the mycelial growth of FON was found abnormal in the VOCs treatment. Overall, mycelial growth inhibition was higher in the strain L3 treatment, the mycelial growth inhibition rate was 19.1% after 5 days incubation (Figure 2b).

### 3.3 Growth promotion of *A. thaliana* with VOCs

**Figure 3:**
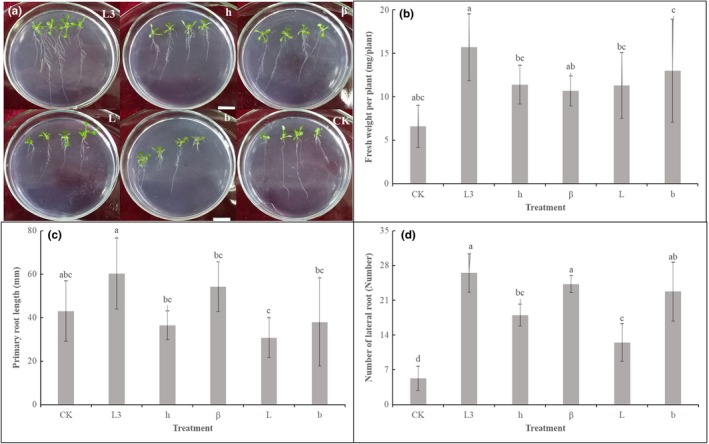
Effect of the VOCs produced by the isolated strains (L3, h, β, L, and b) on the biomass of *Arabidopsis thaliana *Col‐0. (a) Representative photograph showing the effects of VOCs produced by isolated strains. (b) Plant fresh weight, (c) Length of primary root, (d) Number of lateral roots. Lowercase letters above the columns indicate a significant difference at *p* < 0.05

VOCs produced by five antagonistic strains (L3, h, β, L, and b) promoted growth of *A. thaliana *(Figure 3). VOCs emitted by the strain L3 enhanced fresh weights by 2.39‐fold and the number of lateral roots of *A. thaliana* plants by 5.05‐fold, compared to the control. However, no statistically significant effects were found on the primary root length.

### 3.4 Root colonization ability of the strain L3

**Figure 4:**
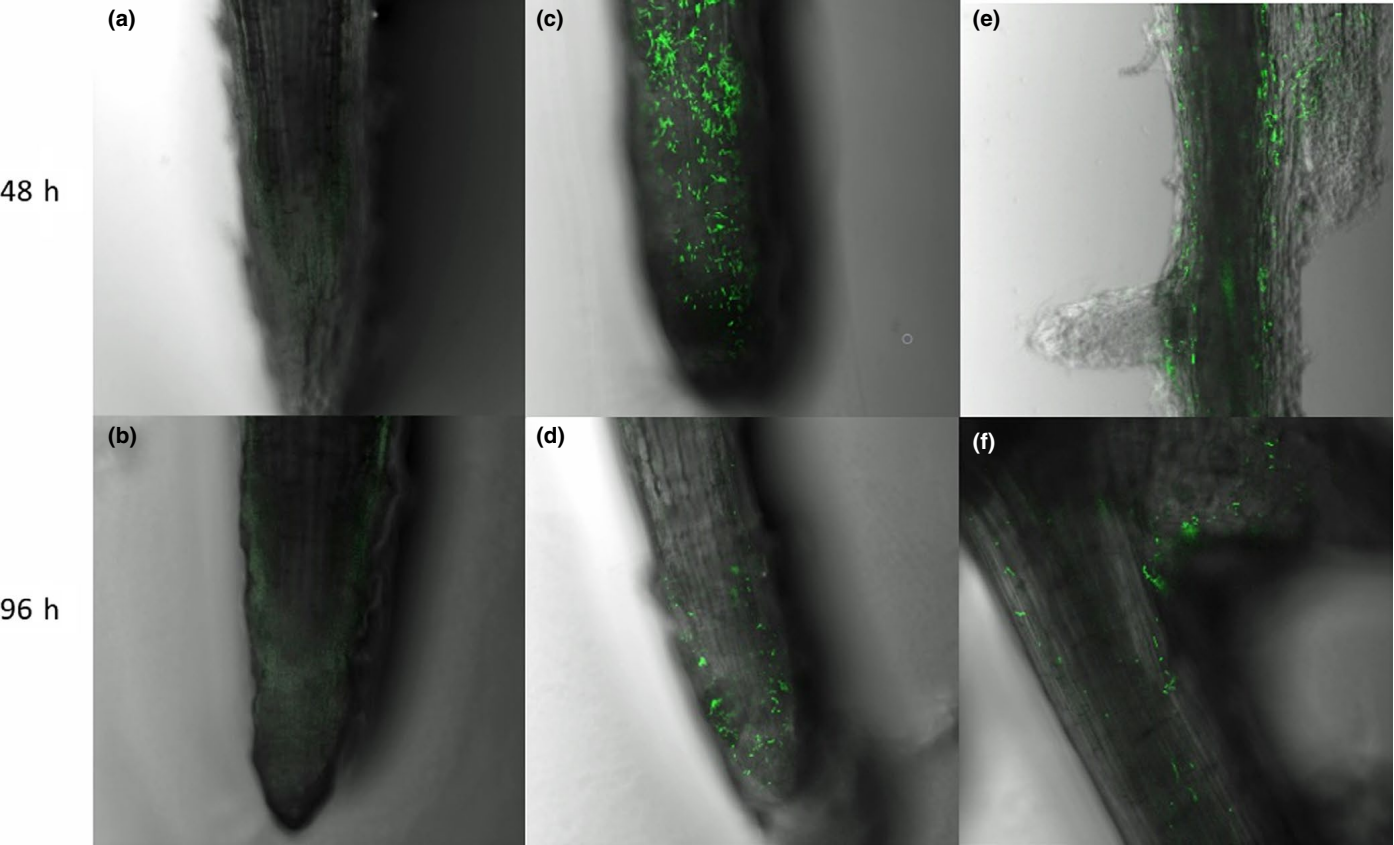
Fluorescence (GFP) micrographs of watermelon roots colonized by GFP‐tagged *Bacillus amyloliquefaciens* L3 in hydroponic system at 48 hr and 96 hr; (a, b) The control, which was not inoculated with GFP‐tagged L3. (c‐f) Root zones of primary root and lateral root junctions colonized by GFP‐tagged L3

**Figure A2:**
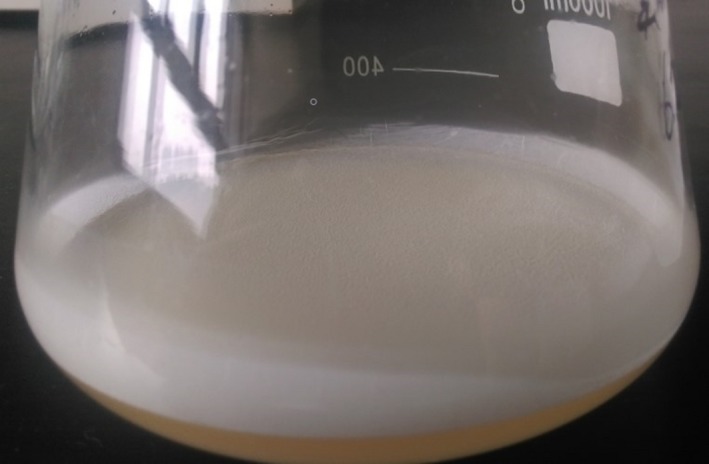
Biofilm formation of the strain L3 in static culture medium (LB)

The strain L3 was found to form thick biofilm in a static culture medium (Appendix Figure A2). In a hydroponic system, the GFP‐tagged cells could easily be distinguished from the background fluorescence in different parts of watermelon roots (Figure 4). The population of the strain L3 colonized on the watermelon's root was approximately 4.65 × 10^6 ^CFU per gram of root after two days of incubation. However, the population of the strain L3 decreased to 1.47 × 10^6 ^CFU per gram of root after 4 days incubation.

### 3.5 Biological control and growth promotion activity of the strain L3

**Figure 5:**
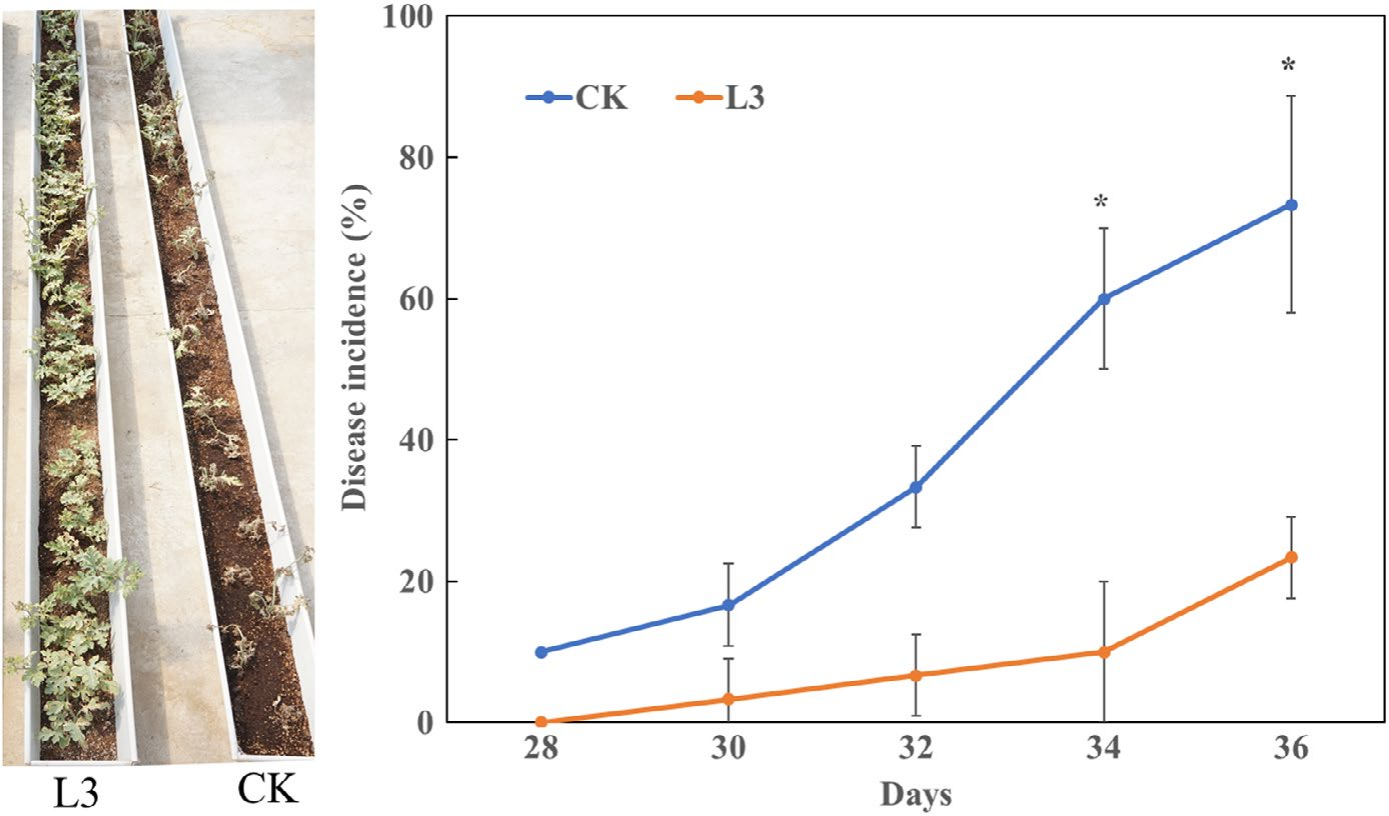
Effect of *Bacillus amyloliquefaciens* L3 on the incidence of *Fusarium* wilt of watermelon in a greenhouse pot experiment. F: control, inoculated with FON, L3: inoculated with FON and the strain L3. “*” above the columns indicate a significant difference at *p* < 0.05

In the greenhouse experiment, the symptoms of *Fusarium wilt* of watermelon appeared 28 days after transplanting watermelon seedlings in the infested substrate and disease incidence increased rapidly during the next 8 days (Figure 5). Disease incidence of the L3 treatment (22.5%) was far lower than the control treatment (71.2%). The relative biocontrol efficiency of the strain L3 was up to 68.4%.

**Figure 6:**
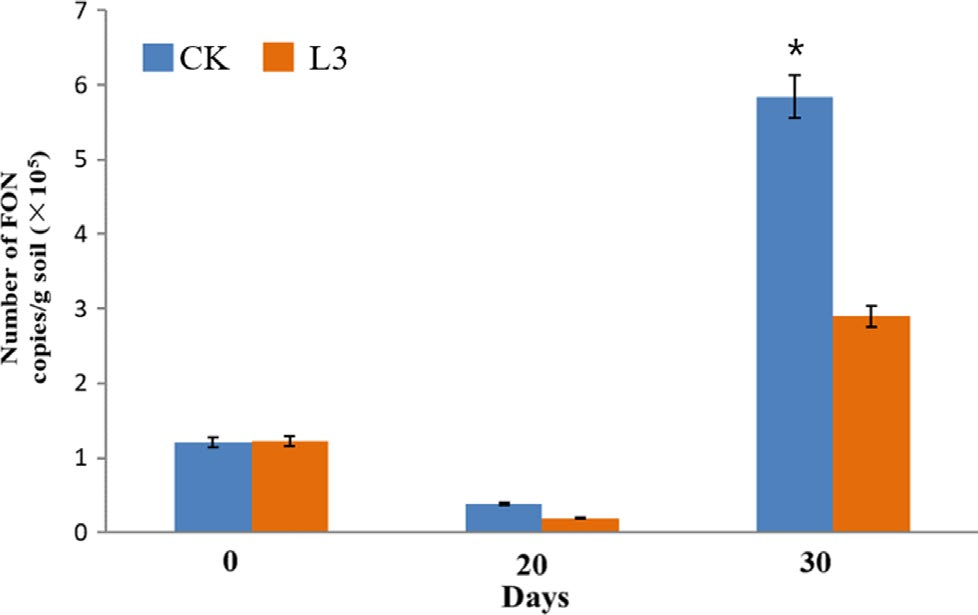
Effect of *Bacillus amyloliquefaciens* strain L3 on the number of FON in the rhizosphere soil of watermelon at 0, 20, and 36 days after transplanting. CK: control, inoculated with FON, L3: inoculated with FON and the strain L3. “*” above the columns indicate a significant difference at *p* < 0.05

Based on real‐time PCR, the population of FON were similar in both treatments during the first 20 days (Figure 6). The populations of FON significantly increased in both two treatments after 36 days of planting in the infested substrate. The population of FON was significantly lower in the L3 treatment (2.90 × 10^5^ copies/g) compared with the CK treatment (5.84 × 10^5^ copies/g). These results corresponded to the disease incidence described above.

**Figure 7:**
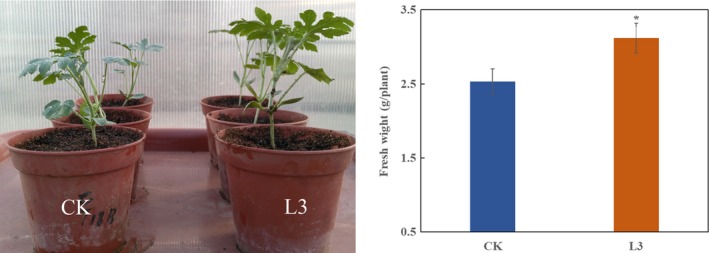
Effect of *Bacillus amyloliquefaciens* strain L3 on the fresh weight of watermelon seedling grown for 20 days in a greenhouse. CK: control treatment, L3: strain L3 was inoculated in the soilless substrate (10^8^ cfu/g). “*” above the columns indicate a significant difference at *p* < 0.05

In the pot experiment, the fresh weights of watermelon plants treated with strain L3 were significantly higher than the control treatment 20 days after transplanting in the non‐infested substrate. The strain L3 treatment showed an increase in 23.4% relative to the control treatment.

### 3.6 Identification of VOCs by SPME‐GC‐MS

**Figure A3:**
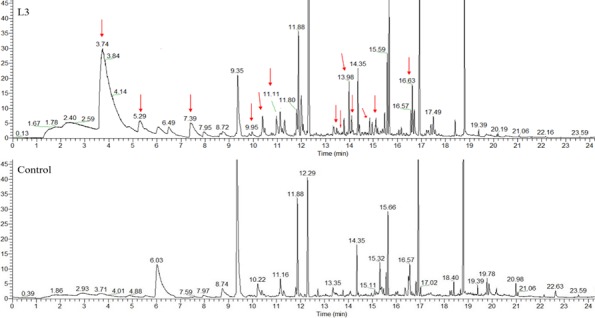
SPME chromatography‐mass spectrometry (GC‐MS) profile of volatile organic compounds (VOCs) produced by the strain L3. Red arrow means the specific VOCs produced by strain L3

Fourteen specific VOCs peaks were identified from the strain L3 (Appendix Figure A3). The identified VOCs included eight ketones (acetoin; 2‐heptanone; 2‐nonanone; 2‐nonadecanone; 2‐undecanone; 2‐dodecanone; 2‐tridecanone; and Bicyclo[2.2.1]heptane, 2,2,3‐trimethyl‐), three alcohols (2,3‐butanediol; 2‐ethyl‐1‐hexanol; and 2‐undecanol), and three alkyls (2,3‐Dimethyldecane, 2,4,6‐trimethyl‐decane, and 2,6,11‐trimethyl‐dodecane).

**Figure 8:**
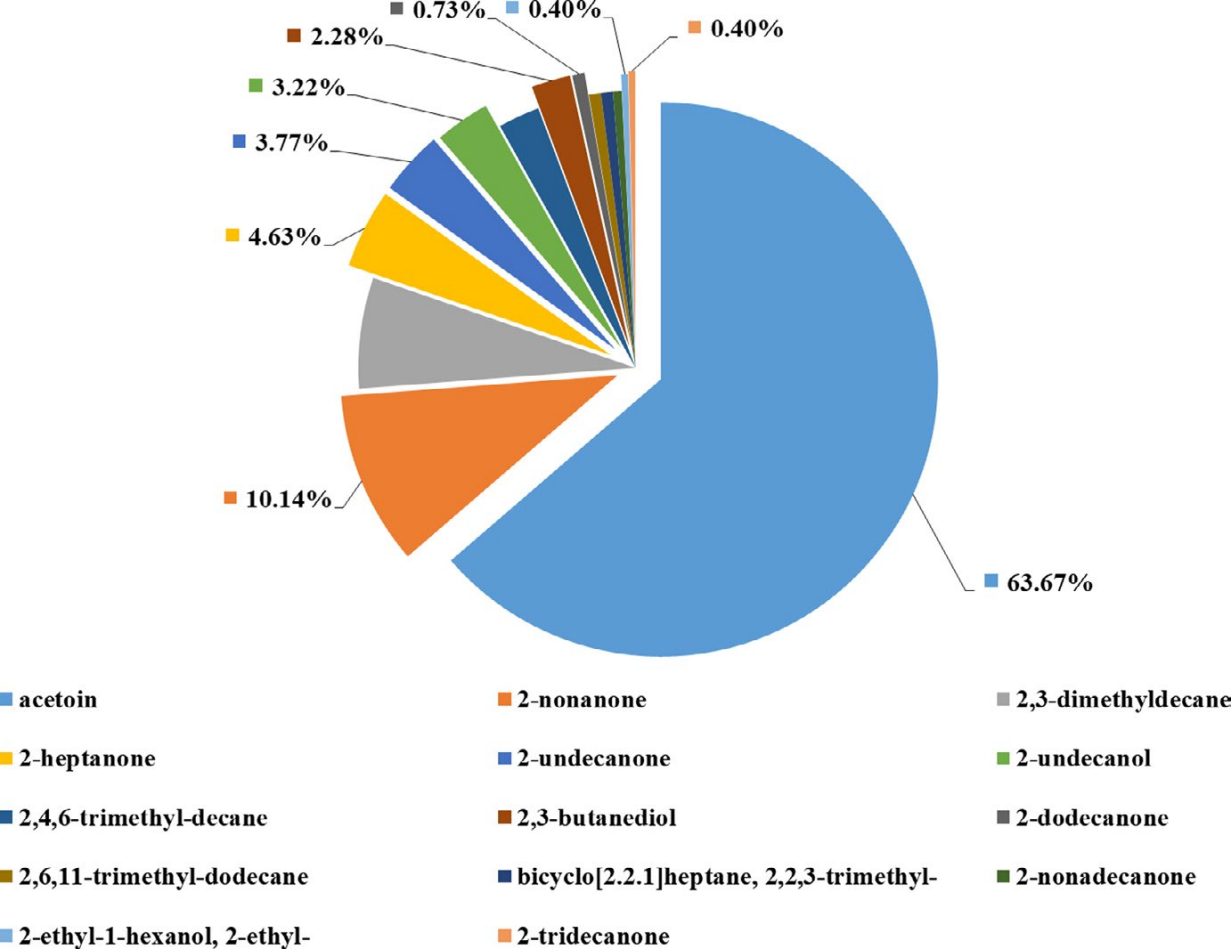
The Relative content of identified volatile organic compounds released by strain L3 based on the peak area of GC‐MS data

Among the 14 identified compounds, nine standard VOCs were purchased for further individual testing of antifungal activities. As indicated by the peak areas of the GC‐MS profile, the relative content order of these nine compounds released by the strain L3 were as follows (Figure 8): acetoin (63.67%), 2‐nonanone (10.14%), 2‐heptanone (4.63%), 2‐undecanone (3.77%), 2‐undecanol (3.22%), 2,3‐butanediol (2.28%%), 2‐dodecanone (0.73%), 2‐tridecanone (0.40%), 2‐ethyl‐1‐hexanol (0.40%).

### 3.7 Verification of standard VOCs against FON

**Figure 9:**
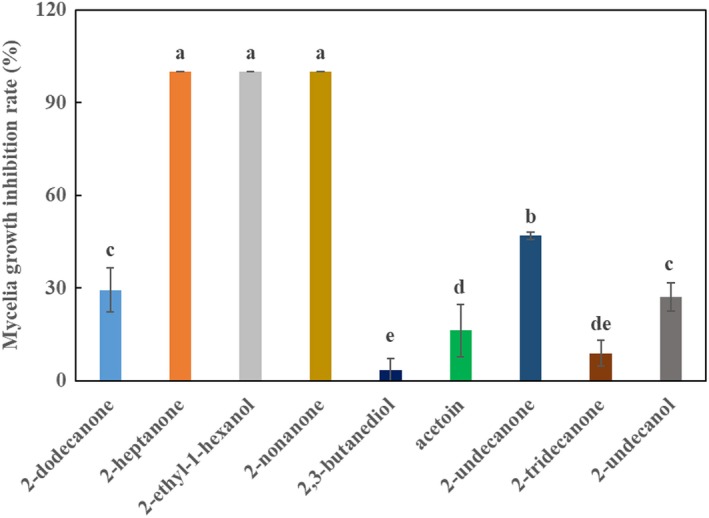
The inhibition of FON mycelia growth by pure VOCs identified from *Bacillus amyloliquefaciens *L3. Lowercase letters above the columns indicate a significant difference at *p* <0 .05

All the nine pure compounds showed antifungal activity against FON; however, the inhibition rates between the nine compounds were significantly different (Figure 9). After four days of incubation, the 2‐heptanone, 2‐ethyl‐1‐hexano, and 2‐nonanone completely inhibited the growth of FON. The inhibition rate of the other six compounds were as follows: 2‐undecanone (46.8%), 2‐dodecanone (29.4%), 2‐undecanol (27.1%), acetoin (16.3%), 2‐tridecanone (8.91%), and 2,3‐butanediol (3.44%).

### 3.8 Plant growth promotion activities of synthetic compounds

**Figure 10:**
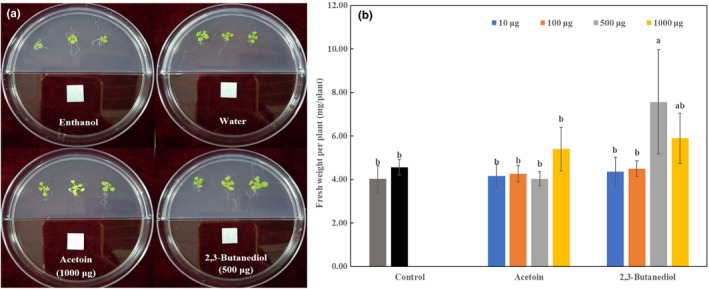
Growth promotion of *Arabidopsis thaliana* Col‐0 with exposure to pure VOCs. (a) Effect of acetoin (1,000 μg/plate) and 2,3‐butanediol (500 μg/plate) on *A. thaliana *Col‐0 growth. (b) Fresh weight of *A. thaliana* Col‐0 after expose to different concentrations of acetoin and 2,3‐ butanediol. Lowercase letters above the columns indicate a significant difference at *p* < 0.05

Of the nine pure compounds, visual inspection revealed that plant shoot and root growth, when compared with the control treatment (water and ethanol), was only enhanced by acetoin and 2,3‐butanediol. The fresh weight of *Arabidopsis* seedlings was increased approximately 1.34‐fold by the presence of acetoin (1,000 μg) and 1.88‐fold by 2,3‐butanediol (500 μg), compared to ethanol treatment respectively (Figure 10).

### 3.9 Identification of strain L3

**Figure A4:**
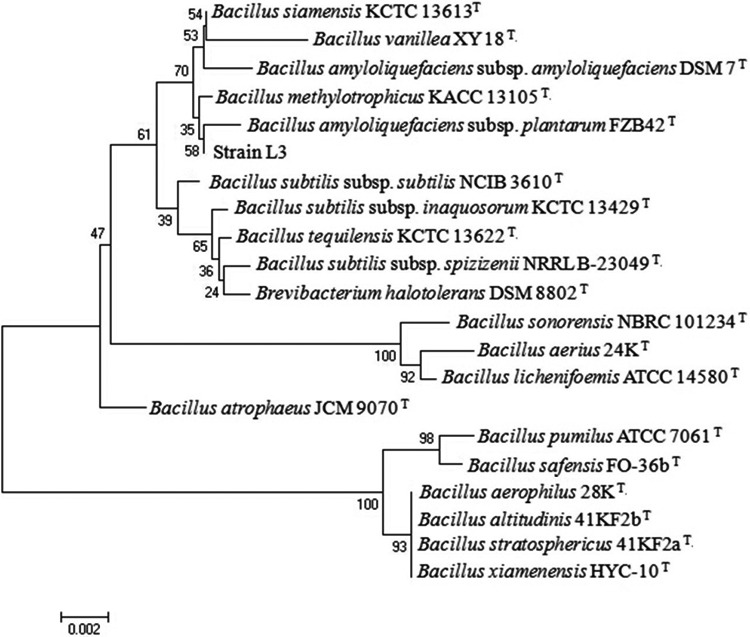
Phylogenetic analysis of strain L3 and related species using the neighbor‐joining approach. Bootstrap values obtained with 1,000 resamplings are indicated as percentages at all branches. The GenBank accession number for each microorganism is shown in parentheses after the species name

Bio‐chemical tests showed that strain L3 was Gram‐positive. Microscopic examination revealed that the strain L3 cells are motile, rod‐shaped, and can form spores when grown on NB culture medium. The phylogenetic tree of the 16S rRNA gene sequencing of strain L3 revealed that it was clustered closely to *B. amyloliquefaciens*, with a sequence similarity score of 99% in NCBI sequence alignment (Appendix Figure A4). Thus, the strain L3 was identified as *B. amyloliquefaciens* after considering all taxonomic characteristics.

## 4 DISCUSSION

The application of biological control agents (BCA) and grafted plants are considered sustainable control approaches to manage Fusarium wilt of watermelon (Ge, Liu, Nwet, & Zhao, 2016; Keinath & Hassell, 2014). Isolation of BCA against phytopathogens, is usually based on initial screening using the dual‐culture plate assay (Hermosa, Grondona, & Iturriaga, 2000). However, the isolation method was mainly considered the ability of the isolated strains to produce diffusible antifungal compounds. Recent studies have demonstrated that VOCs as well as the diffusible antifungal compounds produced by PGPR have equally important functions in antifungal activities (Mao, Chen, & Xia, 2018; Ossowicki, Jafra, & Garbeva, 2017; Xie, Zang, & Wu, 2018). In this study, the dual‐culture and double‐plate chamber method were introduced to distinguish the ability of the isolated strains to produce diffusible and volatile antifungal compounds. Among the strains isolated from the rhizosphere soil samples of watermelon, five strains could produce diffusible and volatile antifungal compounds, though *B. amyloliquefaciens *L3 showed the strongest and consistent antifungal activity against FON.

In addition to the antifungal ability of VOCs, our results clearly showed the plant growth promotion activity of VOCs produced by the isolated strains. Among the five strains, the strain L3 also showed the best plant growth promotion activity. A 2.39‐fold increase in biomass, 1.40‐fold increase in primary root length, and 5.05‐fold increase in number of lateral roots of *A. thaliana* were detected after exposure to the VOCs produced by strain L3. Our results were in accordance with previous reports (Ryu et al., 2003; Vacheron, Desbrosses, & Bouffaud, 2013). However, Park, Dutta, Ann, and Raaijmakers (2015) reported the VOCs released by *P. fluorescens* SS101 increased the biomass of *A. thaliana* up to 8.8‐fold. The proportionately smaller increase in biomass observed in this study could be due to the larger volume of the culture plate and lower concentration of available VOCs to the seedlings in the test chamber. Overall, after considering the antifungal and plant growth promotion activity of the five isolated strains, the strain L3 was chosen for the further analysis.

Biofilm formation and root colonization ability have been recognized as essential factors for BCA to survive in the rhizosphere battlefield against phytopathogens (Bhattacharyya & Jha, 2012; Raaijmakers, Paulitz, & Steinberg, 2009). Based on the fact that the strain L3 could form a thick biofilm in a static culture medium, we expected the strain L3 could ensure good colonization of watermelon roots. Previous studies have shown the elongation and differentiation zones of the plant root, as well as in the lateral roots and the junctions between the roots are preferred by PGPR strains (Zhang et al., 2011). In our study, the GFP signals of GFP‐tagged *B. amyloliquefaciens* L3 were found on the root tips, primary roots, and lateral root junctions (Figure 4). These results are consistent with previous studies (Huang et al., 2012; Ji, Lu, Gai, & Zheng, 2008). Colonization of plant roots by BCA plays an important role in disease control. For instance, Cao et al. (2011) reported that *B. subtilis* SQR9 can provide control of Fusarium wilt of cucumber by root colonizing. Similarly, Li, Zhang, and Zhang (2013) reported that *B. subtilis *HJ5 can provide control of Verticillium wilt of cotton by root colonization and biofilm formation. In this study, we inoculated FON in the soilless culture medium to simulate FON infection, and inoculated *B. amyloliquefaciens *L3 to the rhizosphere of watermelon to prevent the FON infection. We also found *B. amyloliquefaciens *L3 decreased the number of FON that survived in the rhizosphere of watermelon, and the relative biocontrol efficiency was up to 68.4%. Previous results have shown 50% of biocontrol efficacy is acceptable in the pot biocontrol experiment (Minuto, Spadaro, & Garibaldi, 2006; Wang, Yuan, & Zhang, 2013). This evidence indicates that strain L3 is a potential biological control agent to control disease. However, unlike real soil environments, our biocontrol experiment was undertaken in soilless conditions. Different planting media could result in different biocontrol efficiency of the biocontrol agents. Thus, further experiments are needed to explore the biocontrol efficiency of *B. amyloliquefaciens *L3 in field conditions. Previous studies have proved that *B. amyloliquefaciens* can not only suppress Fusarium wilt, but also promote plant growth (Zhao et al., 2014). We found the biomass of watermelons was increased 23.4% by inoculating *B.* *amyloliquefaciens *L3 to the rhizosphere. This result was in accordance with other evidence that have shown plant growth promotion ability of *B.* *amyloliquefaciens* (Chowdhury, Dietel, & Rändler, 2013).

Species of Bacillus have well known versatile weapons for biocontrol of plant pathogens and promotion of plant growth. A variety of mechanisms were employed by Bacillus to fight with phytopathogens and promote plant growth, such as the synthesis of hydrolytic enzymes, antibiotics, hormones, VOCs, and induction systemic resistance of plant (Chowdhury et al., 2015). Bacillus also can produce plant growth promoting hormones and increase nutrient absorption by plants. Among these biocontrol and plant growth promotion mechanisms, less attention has been paid to VOCs. According to recent research, more and more evidence has confirmed the involvement and roles of VOCs in biocontrol and plant growth promotion activities.  Hernández‐León, Rojas‐Solís, and Contreras‐Pérez (2015) reported *P. fluorescens* UM270 could produce dimethylhexadecylamine, a VOC with antifungal and plant growth promotion activities. Pyrazine and benzothiazole released by *B. velezensis* ZSY‐1, inhibits mycelia growth of *Botrytis cinereal *(Yuan et al., 2012). In this study, a total of 14 volatile substances produced by strain L3 were identified. The components of VOCs produced by strain L3 partly overlapped with other *Bacillus* spp., which mainly included alcohol, ketones, and alkanes derivatives (Raza, Wang et al., 2016; Raza, Wei et al., 2016). Among the identified VOCs, 2‐heptanone, 1‐hexano,2‐ethyl‐, and 2‐nonanone exhibited 100% inhibition of mycelial growth of FON. However, 1‐hexano,2‐ethyl‐ was produced in low quantity. Acetoin was the main VOC released by strain L3, but demonstrated low antifungal activity. Considering their antifungal activity and relative peak area, 2‐nonanone and 2‐heptanone were potential candidates as effective antifungal VOCs produced by strain L3.

In previous research, acetoin and 2,3‐butanediol has been used in foods, cigarettes, cosmetics, detergents, and chemical synthesis (Xiao & Lu, 2014). Acetoin is a precursor of 2,3‐butanediol and can be bio‐transformed by plants and microorganisms to 2,3‐butanediol stereoisomers (Javidnia, Faghih‐Mirzaei, & Miri, 2016). In this study, we found that acetoin was the main component of VOCs produced by *B. amyloliquefaciens *L3. 2,3‐butanediol and acetoin, produced by *B. subtilis* GB03 and *B. amyloliquefaciens *IN937, could promote *Arabidopsis* growth and induce systemic resistance (Rudrappa, Biedrzycki, & Kunjeti, 2010; Ryu, Farag, & Hu, 2004). In our study, acetoin and 2,3‐butanediol were the only two VOCs found to promote plant growth. Ryu et al. (2003) reported 2,3‐butanediol could significantly improve the biomass of *A. thaliana* at a concentration of 100 μg/plate. However, a higher concentration (500 μg/plate) used in our study significantly enhanced *A. thaliana *growth. Plant growth promotion regulation by VOCs is dose‐dependent. This different result might be due to the different dilute solvent used in the two experiments, leading to different concentrations of the volatile form of 2,3‐butanediol.

In conclusion, our results demonstrated that *B. amyloliquefaciens *L3 isolated from watermelon's rhizosphere soil could be an excellent candidate for the development of biocontrol agents. We also reported here that VOCs produced by *B. amyloliquefaciens* L3 play important roles in disease control and in growth promoting processes. However, further studies are still needed to understand the role of VOCs produced by *B. amyloliquefaciens* L3 under field conditions.

## CONFLICT OF INTERESTS

The authors declare that there is no conflict of interests.

## AUTHORS CONTRIBUTION

YC Wu and Y Ma designed and supervised the study. CG Li was involved in the greenhouse pot experiment. JY Zhou was involved in verifying plant growth promotion activities of VOCs. All of the other experiments were performed by YC Wu.

## ETHICS STATEMENT

None required.

## Data Availability

All data are provided in full in the results section of this article. The raw sequence data have been deposited in NCBI GenBank under the accession number MK394005.

## APPENDIX 1

**Table A1:** Pure compounds purchased from reagent companies

Compound Name	Chemical structure	Source	Purity (%)
Acetoin		A	≥96
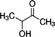
2,3‐Butanediol		A	≥96
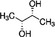
2‐Heptanone		A	≥98
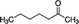
1‐Hexanol, 2‐ethyl‐		A	≥99
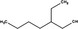
2‐Nonanone		A	≥96
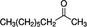
2‐Undecanone		A	≥98
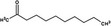
2‐Undecanol		A	≥97.0
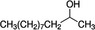
2‐Dodecanone		A	≥97.0
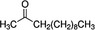
2‐Tridecanone		A	≥96.0
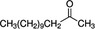

Note:

A: Sigma‐aldrich LLC.
